# Parliament and the Professions

**Published:** 1920-01-03

**Authors:** 


### PARLIAMENT AND THE PROFESSIONS.

### Hemel Hempstead Military Hospital.

Mr. Churchill stated that a recent inspection of the hospital revealed that the administration was seriously at fault, and the supersession of the late commanding officer was recommended by the inspecting officer. At a previous inspection, in August, everything had been found satisfactory.

### Young Men's Christian Association.

It was stated that advances have been made by the War Office, with Treasury approval, to the Young Men's Christian Association in respect of exceptional calls upon their resources. These sums are repayable with interest at 5 per cent.

### Death Certification.

Mr. J. Davison asked the Minister of Health whether, in connection with his consideration of intended reforms in the present law of death certification, he will take note of the desirability of providing that no medical practitioner shall certify a death without having first inspected the body, and that all deaths occurring suddenly after operations, or vaccination, or inoculation, or drug injections, shall be reported to the coroner by the medical practitioner in attendance. Dr. Addison said that the point referred to in the question shall be considered.

### Orthopædic Hospitals.

Captain Ormsby-Gore, in a question to the Minister of Health, alluded to proposals by Sir Robert Jones and Dr. Girdlestone that open-air country orthopædic hospitals for children suffering from rickets, infantile paralysis, and surgical tuberculosis should be established. Dr. Addison said that this interesting scheme is under consideration and concerns a very important one of those special institutional services the development of which is greatly needed, and which are now under the direct consideration of the Consultative Councils, who have been asked to advise as to a scheme of medical and allied services generally.

### Diphtheria and Scarlet Fever.

Dr. Addison said that pressure for civilian needs recently was due to the non-return of fever establishments of the Metropolitan Asylums Board by the military authorities. The dimensions of the present scarlet fever epidemic are smaller and the maximum figure is a little later in the season than in the two previous epidemics for 1907 and 1914. Figures indicated that both for scarlet fever and diphtheria the maximum has been passed.

### Administration of Anæsthetics.

Mr. Gilbert alluded to recent inquests when it was proved that anæsthetics had been administered in hospitals by unqualified persons, and Dr. Addison stated that the subject is receiving attention in relation to the recommendations of the Departmental Committee on Coroners.

### National Relief Fund.

The Prime Minister stated that the use of the National Relief Fund for exceptional distress due to unemployment is now being considered by the Committee of the Fund, and an announcement on the subject may be expected shortly.

### The Position of Voluntary Hospitals.

Mr. Lyle asked the Prime Minister whether he could state on behalf of the Government that there would be no attempt to nationalise the great voluntary hospitals; and whether he was aware that many such institutions were suffering at the present moment on account of charitable people holding back owing to uncertainty as to the Government's intentions. The Minister of Health (Dr. Addison) said that, as he had already stated, the Government have no such intention.

### Mortality in India during 1918.

The Secretary of State for India stated that the number of reported deaths in British India under each principal heading during 1918 was as follows:

| | |
|---|---|
| From cholera ... ... ... ... ... ... | 560,802 |
| ,, smallpox ... ... ... ... ... ... | 93,076 |
| ,, plague ... ... ... ... ... ... | 440,752 |
| ,, fever ... ... ... ... ... ... | 11,134,441 |
| ,, dysentery and diarrhœa ... ... | 276,648 |
| ,, respiratory diseases ... ... ... | 430,935 |
| ,, injuries ... ...... ... ... | 98,969 |
| ,, all other causes ... ... .. ... | 1,861,178 |
| Total ... ... ... | 14,895,801 |

No deaths are reported as being due to famine. Influenza cases are not tabulated separately.

### Nurses' Registration.

The following Acts of Parliament received the Royal Assent on December 23, 1918:—

Nurses' Registration Act, 1919.
Nurses' Registration (Scotland) Act, 1919.
Nurses' Registration (Ireland) Act, 1919.

